# Injection of concentrated growth factors from plasma for treating large chronic lower-extremity ulcers defects

**DOI:** 10.3389/fbioe.2025.1681705

**Published:** 2025-11-28

**Authors:** Zhiqiang Zhang, Nan Cao, Chaoqi Zuo, Xuan Guo, Yilei Wu, Yongxia Liu, Guoyan Liu, Xueqing Wang, Shengji Zhou, Haixia Wang, Guangliang Zhang

**Affiliations:** 1 Dermatology Hospital of Shandong First Medical University, Jinan, Shandong, China; 2 Shandong Provincial Institute of Dermatology and Venereology, Shandong Academy of Medical Sciences, Jinan, Shandong, China

**Keywords:** concentrated growth factors, chronic ulcers, wound healing, tissue engineering, regenerative medicine

## Abstract

The treatment of chronic lower-extremity ulcers is challenging, with a high ulcer recurrence rate.In chronic wounds, the healing process is stagnated in the inflammatory phase, characterized by persistent activation of the innate immune response. Compared with normal wounds, its environment is highly changed, with the downregulation of growth factor (GF) receptors because of resident low-mitogenic cells. Recently, the use of autologous growth factors has been shown to improve wound healing. We used concentrated growth factors extracted from plasma to treat chronic lower-extremity ulcers. The patients were treated with injections of concentrated growth factors extracted from autologous blood plasma. The wound condition was evaluated five days after each injection. The five patients received five injections of concentrated growth factors once a week. After two weeks, the area of the wound reduced, and the lipodermatosclerosis and abnormal pigmentation significantly improved. The wound was fully epithelialized after treatment. Injection of concentrated growth factors extracted from plasma may effectively treat chronic lower-extremity ulcers. However, the sample size may be relatively small, and we plan to continue collecting additional cases to minimize potential bias.

## Introduction

The skin, situated in the outermost layer of the human body, comprises approximately 15% of the total body weight. It is a vital and effective barrier for protecting the body from environmental elements, maintaining internal homeostasis, and facilitating various physiological functions (Abuhamad et al., 2024). When the skin is injured and forms a wound, the body initiates a process to restore this natural protection (Jang et al., 2021). Wounds include two major types: acute and chronic wounds. Chronic wounds, also known as ulcers, do not heal spontaneously within 4 weeks and are accompanied by a persistent inflammatory state (La Monica et al., 2024). Chronic wounds fail to progress through the normal phases of healing in an orderly and timely manner (Grada and Phillips, 2022). The common causes of chronic lower extremities wounds include cardiovascular diseases, diabetes, and pressure (Franz et al., 2024). Because they cause disability that requires long-term care from skilled professionals, chronic wounds have a significant socioeconomic impact on patients (Rodriguez-Valiente et al., 2024). It is estimated that 1%–2% of the world’s population will be affected by chronic wounds at least once in their lifetime (Jarbrink et al., 2016).

In chronic wounds, the healing process is stagnated in the inflammatory phase, characterized by persistent activation of the innate immune response (Versey et al., 2021). An increasing level of reactive oxygen species and metalloproteases (MMPs) that cause both GF decrease and extracellular matrix (ECM) degradation has been found in chronic wounds (Diegelmann, 2003; Blanco-Fernandez et al., 2021). This decreased availability of cytokines and growth factors, along with reduced microcirculation and infections by multidrug resistant and biofilm-forming microbes, are critical factors that lead to the development of chronic nonhealing wounds (Sun et al., 2020).

The progression of these conditions not only further damages the wound and worsens the infection but also increases the risk of tissue necrosis (Zhao et al., 2016). Ultimately, the wound persists in healing for an extended period and may even require amputation, which seriously impacts the patient’s quality of life (Song et al., 2024).

Traditional treatment includes surgical and nonsurgical methods, such as topical dressings, medicated formulations, and skin grafts, which promote the self-healing process of the wound (Abuhamad et al., 2024). However, these traditional methods have limited effects on the healing of chronic wounds because of the significant delay and deficiency of the healing process (Abuhamad et al., 2024). The current therapeutic strategies employed by hospitals fail to address the imbalance of proteolytic activity, leading to prolonged hospital stays and huge costs. Therefore, exploring therapeutic alternatives to restart the healing process by focusing on the specific mechanisms involved in the healing process of chronic wounds is necessary (La Monica et al., 2024).

Anti-inflammatory and antioxidant processes should be used concurrently to change the reducing and hypoxic chronic wound bed into a microenvironment that allows wound healing. In addition, cell proliferation, angiogenesis, and ECM molecule accumulation must be stimulated to promote wound closure and scar formation (La Monica et al., 2024). Therefore, to promote chronic wound healing, the first step is to reduce the inflammation and stimulate infiltration by keratinocytes, fibroblasts, and endothelial cells for ECM synthesis and remodeling during wound healing (Smith and Rai, 2024). Therefore, addressing the deficiency of growth factors in the peri-wound microenvironment could represent a promising therapeutic strategy for chronic wound management.

A concentrated growth factor (CGF) is a third-generation platelet concentrate harvested by variable-speed centrifugation of plasma. In 2006, Sacco pioneered the development of Concentrated Growth Factor (CGF). Compared to the preparation processes of PRP and PRF, CGF production is characterized by the absence of anticoagulants and the use of variable-speed centrifugation. This unique protocol results in a denser three-dimensional fibrin scaffold than those of PRP and PRF, thereby providing superior flexibility and tensile strength. Additionally, it enables a more gradual release of growth factors, ultimately enhancing its capacity for tissue regeneration and anti-inflammatory activity (Mijiritsky et al., 2021). In a study by Huang et al. on a mouse model of full-thickness skin defect, histological analysis revealed significantly higher expression of vascular endothelial growth factor and CD34 in CGF-treated wounds compared to the PRP group (Huang et al., 2022). This indicates that CGF has a more pronounced effect on promoting wound healing than PRP. Over the past two decades, CGF has emerged as a promising therapeutic option to facilitate wound closure and promote tissue regeneration (Pensato et al., 2024). However, exploring the optimal CGF application method to effectively reduce wound size remains a critical and understudied aspect of wound care. The current study aims to explore the clinical effects of CGF injections on wound healing and provide real-world evidence to address this knowledge gap by investigating the relationship between the number of CGF applications and wound size reduction in chronic wounds. In this study, we aim to refine CGF therapy to enhance its efficacy in promoting chronic wound healing.

## Methods and Results

This is an open-label, single-arm controlled trial conducted at a single dermatology care center in the China (XXMZR-20240607-002). The Institutional Review Board of our Hospital approved this study conducted in accordance with the Helsinki Declaration guidelines. All eligible patients provided written informed consent prior to enrollment.

### Study population and inclusion and exclusion criteria

The inclusion criteria for the patient records analyzed were as follows: 1. Nonhealing wound of more than 1 month; 2. Pathological examination of the wound showed a chronic ulcer; and 3. The area of the wound was too large to be sutured directly. The exclusion criteria were the following: 1. Tumor wound; 2. Acute wound; 3. Bacteremia, sepsis, thrombocytopenia, or other hematological disease; and 4. The physical condition was too poor to receive debridement. Photographs were taken during the last follow-up.

### Study design and interventions

This study was conducted from June 2023 to October 2024 in a single tertiary dermatology center. The institute (hospital) is located in Jinan, known as the “Spring City,” and provides high-quality medical services to over 500,000 outpatient visitors and 5,000 inpatient cases annually. It has earned a distinguished reputation, ranking among the top ten in China according to the Fudan Medical Specialty Reputation Rankings. Before the surgery, a pathological examination of the wound was performed to confirm the diagnosis of a chronic ulcer. General or local anesthesia was used in all the cases.

### Extraction of CGF in gel phase from the patient’s blood samples

To obtain the CGF to be applied to the ulcer, a sufficient volume of venous blood was drawn from each patient according to the area of the wound. A wound with an area of 10 cm^2^ required one tube of blood. The patient’s blood was centrifugated using centrifuge tubes supplied by the manufacturer to obtain the plasma (a tube with anticoagulant on the wall and a green cap).

For centrifugation, 9 mL of the patient’s blood was harvested with a vacutainer tube provided by the manufacturer. After blood collection, the tubes were centrifugated in a Medifuge centrifugal accelerator (Silfradent, Italy). The parameters were set as follows: 30 s of acceleration to 2,700 rpm; 2,700 rpm for 2 min; 2,400 rpm for 4 min; 2,700 rpm for 4 min; 3,000 rpm for 3 min; and 36 s of deceleration and rotation to stop.

After centrifugation, the blood in the tubes was divided into three layers. The top, middle, and bottom layers were 2 mL of platelet-poor plasma, CGF, and red blood cells, respectively. Furthermore, we measured the concentrations of VEGF, PDGF, and TGF in CGF using ELISA and found that the concentrations of VEGF and PDGF-BB in CGF were significantly higher than those in the top platelet-poor plasma (PPP) (Supplementary Figure S1). Thus, the middle layer was harvested for application. Approximately 2.5 mL of liquid CGF can be harvested from the tube (Figure 2A).

### Surgical technique

**FIGURE 1:**
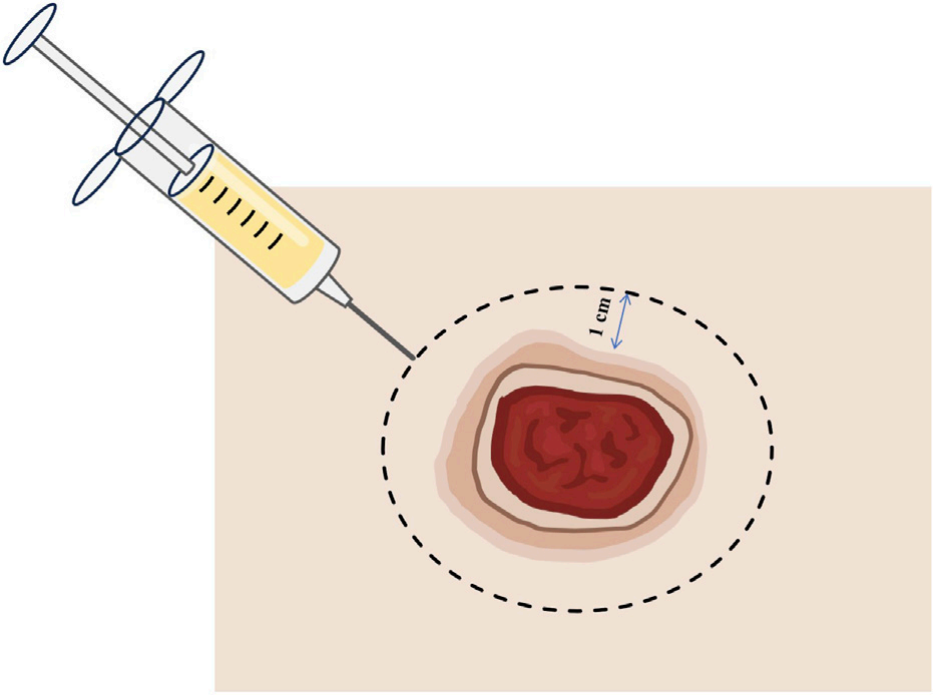
Schematic diagram of injection of CGF on treatment of ulcer wound.

**FIGURE 2:**
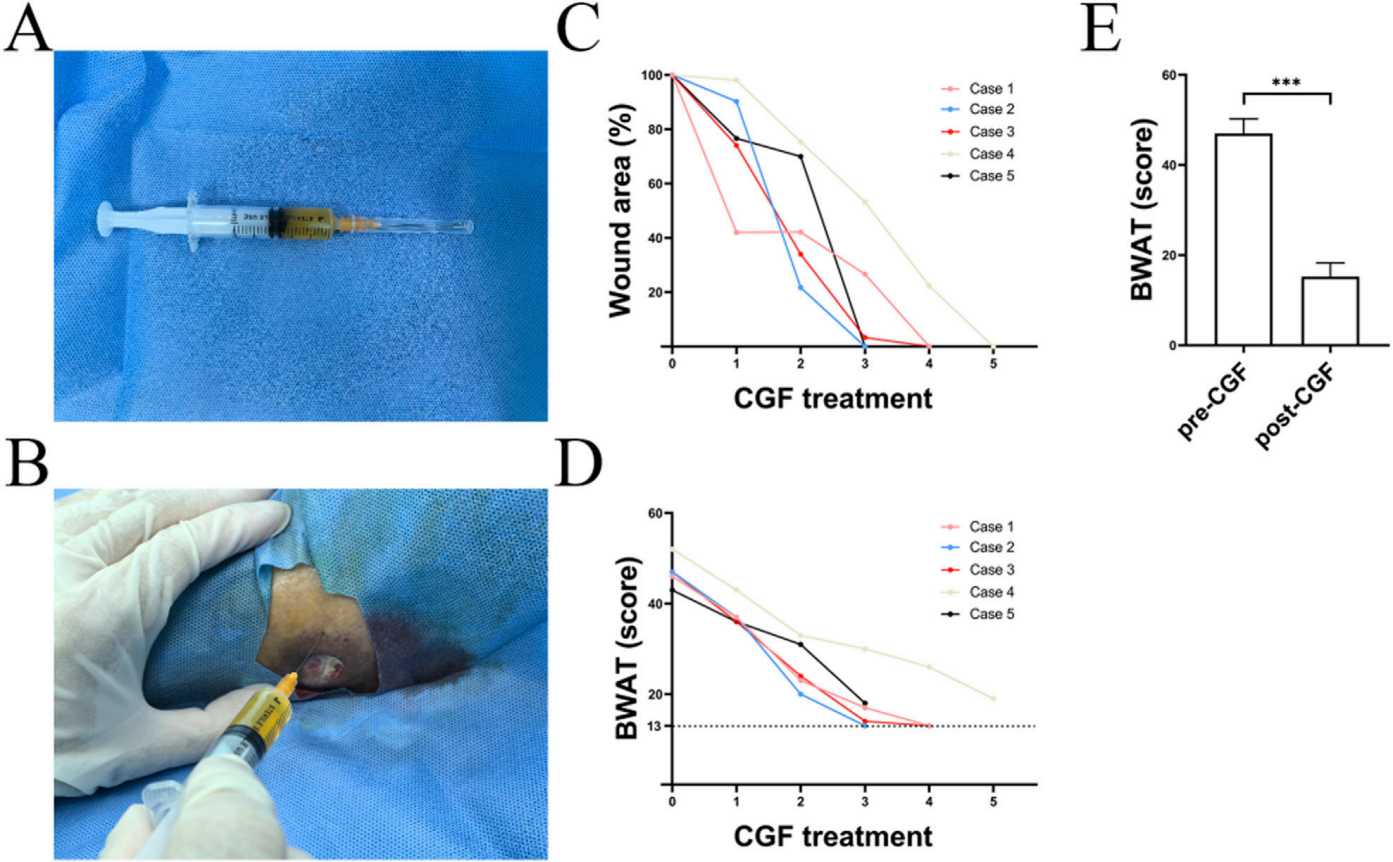
Effects of CGF injection on the treatment of an ulcer **(A)** Harvest of CGF, **(B)** Process of CGF injection, **(C)** Wound area, **(D)** BWAT score, **(E)** Statistical analysis of BWAT scores.

After complete blunt debridement, the skin adjacent to the wound was injected with CGFs extracted from the patient’s plasma. The distance between the injection point and the edge of the wound was 1 cm. The distance between the two injection points was 0.5 cm (Figures 1, 2B). The volume of each injection is 0.1 mL. Finally, Vaseline gauze and gauze dressings were used to cover the wound.

The patient received the first dressing change 3 days after the CGF injection. The dressings were then changed every 2 days. Moreover, the skin adjacent to the wound was injected with CGF weekly until the wound was healed, and antibiotics were administered for 2 weeks.

### Data collection and analysis

To analyze the main variable of the study (area of the ulcer), the ulcer was photographed at the start and end of treatment and the results were analyzed using ImageJ. The wound condition was measured at the start and end of treatment using the Bates-Jensen wound assessment tool (BWAT). Patient satisfaction was assessed via self-evaluation using a visual analog scale (on a 10-point scale). Comparative analysis was performed using contingency tables with the paired t-test. P < 0.05 was considered statistically significant. Data were analyzed using IBMSPSS (SPSS, Inc., Chicago, IL, USA).

### Patient demographics

Five patients, including four women and one man, were included in this study. They were aged 13 to 66, with an average of 43.8 years. The histopathological findings showed no evidence of tumor cells in all five patients. Chronic inflammation, allergic vasculitis, and stasis dermatitis were diagnosed in two, one, and two cases, respectively. The duration of the wound was from 2 to 12 months, with an average of 5 months (Table 1).

**TABLE 1:** Patient demographic characteristics.

Case	Sex	Extremity	Area of ulcer (cm^2^)	Causes	Number of injection	BWAT (scores)	
						Before treatment	After treatment
1	F	R	8.0	Chronic inflammation	4	46	13
2	M	L	43.5	Allergic vasculitis	3	47	13
3	F	L	11.0	Stasis dermatitis	4	47	13
4	F	L	9.5	Chronic inflammation	5	52	19
5	F	L	2.5	Stasis dermatitis	3	43	18

F, female; M, male; R, right; L, left.

### Clinical outcomes

Complete wound healing was achieved in all patients (5/5) during the first 6 weeks of observation: in 2 (40%), 2 (40%), and 1 (20%) in 4, 5, and 6 weeks, respectively (Figure 2C). The mean healing time was 4.8 weeks.

The BWAT score before treatment ranged from 43 to 52 (47 ± 3.240). The score at the last follow-up ranged from 13 to 19 (15.2 ± 3.033). There were significant differences in the scores between the two groups of data (<0.01) (Figures 2D,E), suggesting that CGF injections can effectively promote wound healing (Table 1). After 2 weeks, the area of the wound reduced, and the lipodermatosclerosis and pigmentation significantly improved. No complications or undesirable effects of CGF (such as skin irritation, pain, or allergic reactions) were observed in any of the patients.

### Case descriptions

**FIGURE 3:**
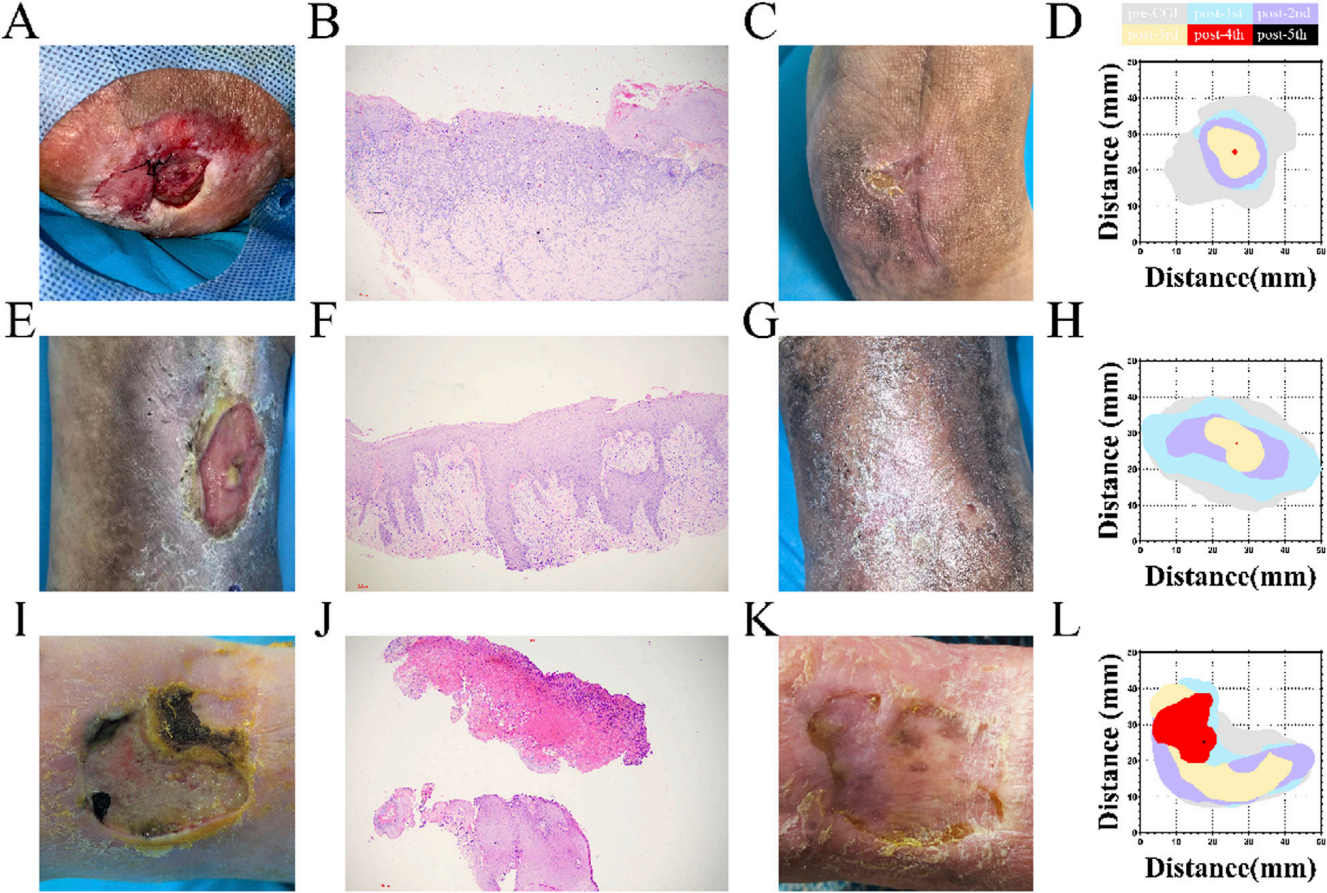
Treatment of cases **(A,E,I)** Wound condition before treatment of patients 3, 1, and 5; **(B,F,J)** Pathological results of the ulcer in patients 3, 1, and 5; **(C,G,K)** Wound condition in patients 3, 1, and 5 at 3 months following treatment; **(D,H,L)** Quantification of the wound area during the treatment of patients 3, 1, and 5.

Patient 1 was involved in a car crash 30 years ago after which her right ankle was fused. She recently developed an ulcer on the heel. After 6 months, the area of the ulcer was 8.0 cm^2^, with muscle exposure and lipodermatosclerosis around the wound. A stealth tunnel with an area of 3.0 cm × 1.5 cm was detected (Figure 3A). Patient 3 underwent superficial vein ablation of left lower limb veins for varicose veins of the lower limb of 20 years duration. She developed an ulcer above the ankle 1 year ago. The area of the ulcer was 11.0 cm^2^, with exposure of the superficial fascia, lipodermatosclerosis, and abnormal pigmentation around the wound (Figure 3E). Patient 4 was diagnosed with eczema with swelling of the left lower limb 44 years ago. She developed an ulcer on the dorsum of her left foot 3 months ago. The area of the ulcer was 9.5 cm^2^, with exposure of the deep fascia (Figure 3I). Pathological examination revealed hyperkeratosis, exudation, and thickening of the spinous layer of the skin around the lesion, and no tumor cells were found in these three cases (Figures 3B,F,J). The wound was epithelialized after complete blunt debridement and CGF injections in Patient 1 (Figures 3C,D), Patient 3 (Figures 3G,H) and Patient 4 (Figures 3K,L).

## Discussion

Chronic ulcers of the lower limb are challenging to manage in clinical practice. They have a long course, are difficult to cure, and easily relapse, which seriously affects patients’ quality of life. A decrease in the local concentration of GFs, tissue ischemia, hypoxia, and wound infection are always present in these ulcers (Joorabloo and Liu, 2024). These factors lead to an imbalance in the ECM composition and a decrease in the production of fibroblasts. Fibroblast dysfunction can prevent wound healing and epithelialization. Therefore, the change in the microenvironment is the key factor associated with difficulty in wound healing. In addition, the positive bacterial culture rate in these ulcers is greater than 80%, which makes wound healing more difficult. The average healing time ranges from 6 to 12 months, and approximately 20% of wounds that are appropriately treated do not heal within 24 months (Aleksandrowicz et al., 2021).

CGF, a third-generation platelet concentrate, was first developed by Sacco (Akcan and Unsal, 2020) and is harvested by variable-speed centrifugation of plasma. Additionally, it has a high concentration of GFs. Masuki et al. assessed the levels of GFs and cytokines in CGF using ELISA and found that the proportion of platelet-derived GF (PDGF), transforming growth factor, vascular endothelial growth factor (VEGF), insulin-like growth factor-1 (IGF-1), and epidermal growth factor in the concentrate was high (Masuki et al., 2016). Kao reported on CGF membrane treatment with CGF produced from autologous venous blood. The treatment was applied to chronic skin wounds in seven patients with full-thickness soft tissue defects. All the patients achieved satisfactory results after 6–32 weeks of treatment. During the treatment process, a liquid nitrogen cryospray was used to inhibit the overgrowth of granulation tissue until complete re-epithelialization was achieved (Kao, 2020). Kabilamurthi et al. explored the effectiveness of CGF in wound healing after dental implant placement procedures. They found that compared to the control group, the wounds in the ten patients who received the CGF membranes before suturing healed in less time than those of the others (Kabilamurthi et al., 2021). Liu et al. reported that an ulcerated chronic wound on the lower leg with an area of 2.0 cm × 3.5 cm was treated with CGF gel, and the wound healed well after three episodes of CGF treatment. All these reports verify the effectiveness of the CGF gel in promoting wound healing (Liu et al., 2021).

In our study, the patients had larger skin defects. Therefore, we employed a different method of using CGF from that reported in previous studies. Liquid CGF was injected away from the wound into the skin adjacent to the wound. This approach promoted healing by modifying the local microenvironment of the wound edges, while simultaneously avoiding potential inefficacy caused by direct injection into infected areas. And we believed that in this method, the growth factors could work directly on fibroblasts, mesenchymal stem cells, and endothelial cells. It could promote quick cell proliferation, collagen synthesis, and angiogenesis, resulting in rapid wound healing. Proteins, such as PDGF and bFGF, in the CGF act as chemotactic factors and attract cells from the surrounding skin tissue into the wound, thereby achieving cell migration (Tabatabaei et al., 2020). Therefore, CGF injection may also be an effective method of treating wounds.

In these cases, combining CGFs and antibiotics resulted in the healing of large infected wounds. The stealth tunnel also closed within 2 weeks after treatment. During wound healing, the epidermis and dermis extended simultaneously, and there was no hyperplasia of granulation tissue to prevent epithelialization (Supplementary Figure S2). Deep wounds with muscle exposure and lipodermatosclerosis may also be treated by CGF injections. To the best of our knowledge, our study described for the first time that the injection of CGF extracted from plasma can be used to treat large chronic lower-extremity ulcers.

## Limitations

Although the preliminary data on the effects of CGF injections are encouraging, there are several unanswered questions. The required concentration and dosage of CGF need to be further investigated. Tabatabaei et al. reviewed 45 articles published on the use of CGFs and found that different concentrations of CGFs among the donors had different effects on CGF treatment outcomes (Kabilamurthi et al., 2021). In addition, the maximum area of an ulcer that can be healed by CGF injections without skin grafting needs to be explored.

## Conclusion

The management of chronic ulcerative wounds of the lower extremities poses a significant challenge due to their insufficiency of blood supply and high recurrence rates. CGFs extracted from plasma may be effective for treating chronic lower-extremity ulcers. The procedure appears to be safe and feasible for popularization and application.

## Data Availability

The original contributions presented in the study are included in the article/Supplementary Material, further inquiries can be directed to the corresponding author.

## Supplementary material

SUPPLEMENTARY FIGURE S1 Concentrations of growth factors (TGF-β1, PDGF-BB, VEGF) in top platelet-poor plasma (PPP) and CGF preparations.

SUPPLEMENTARY FIGURE S2 Wound condition before treatment **(A, B)**, after the first injection of CGF **(C)**, after the second injection of CGF **(D)**, after the third injection of CGF **(E)**, and after the fourth injection of CGF **(F)**.
