# Supplementary figures and images for: Injection of concentrated growth factors from plasma for treating large chronic lower-extremity ulcers defects
Source: Front Bioeng Biotechnol. 2025 Nov 28;13:1681705. doi: 10.3389/fbioe.2025.1681705 (PMC12698627; doi:10.3389/fbioe.2025.1681705)

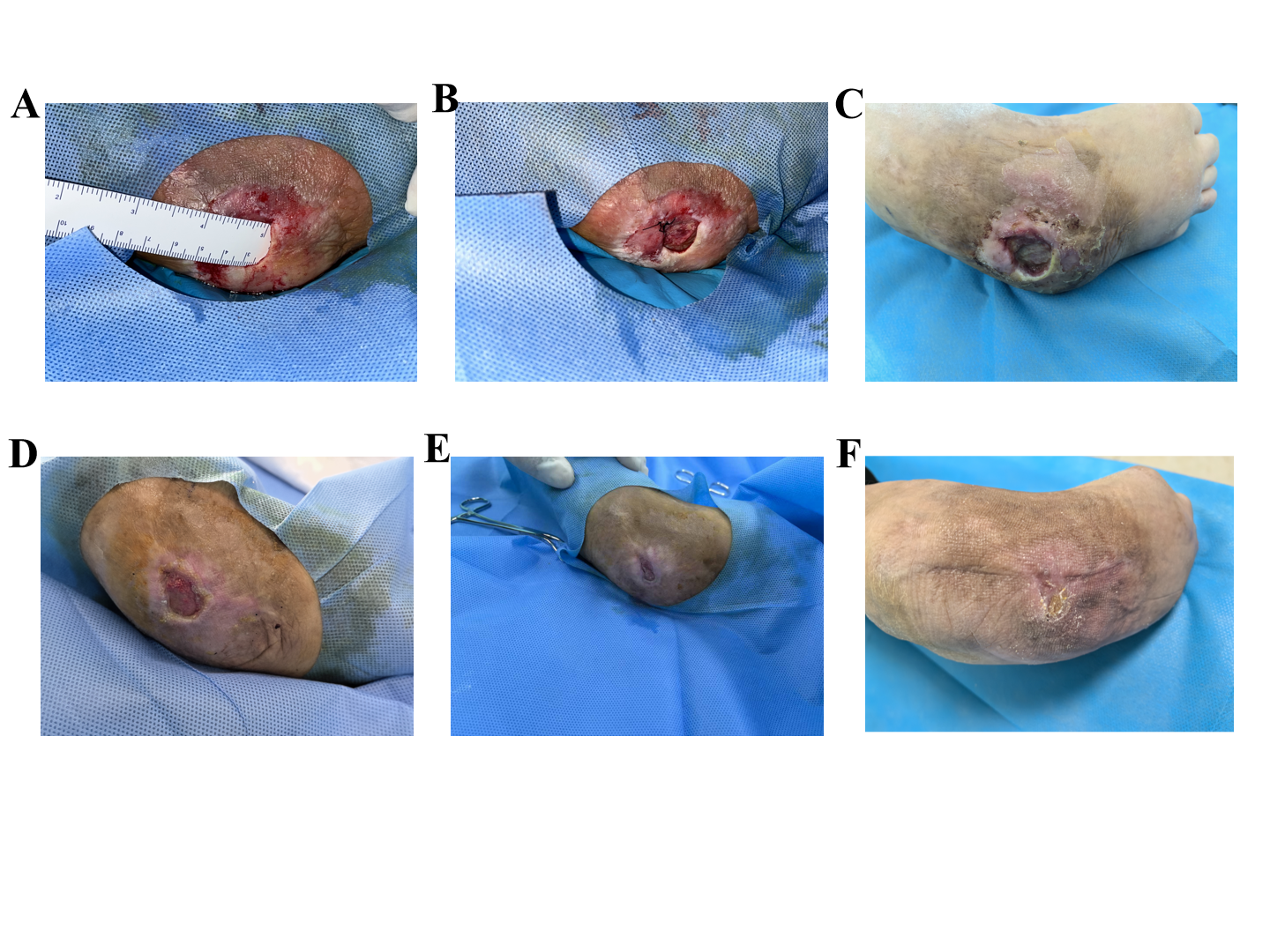

Supplement: Supplementary file 1 [file Image2.tif]

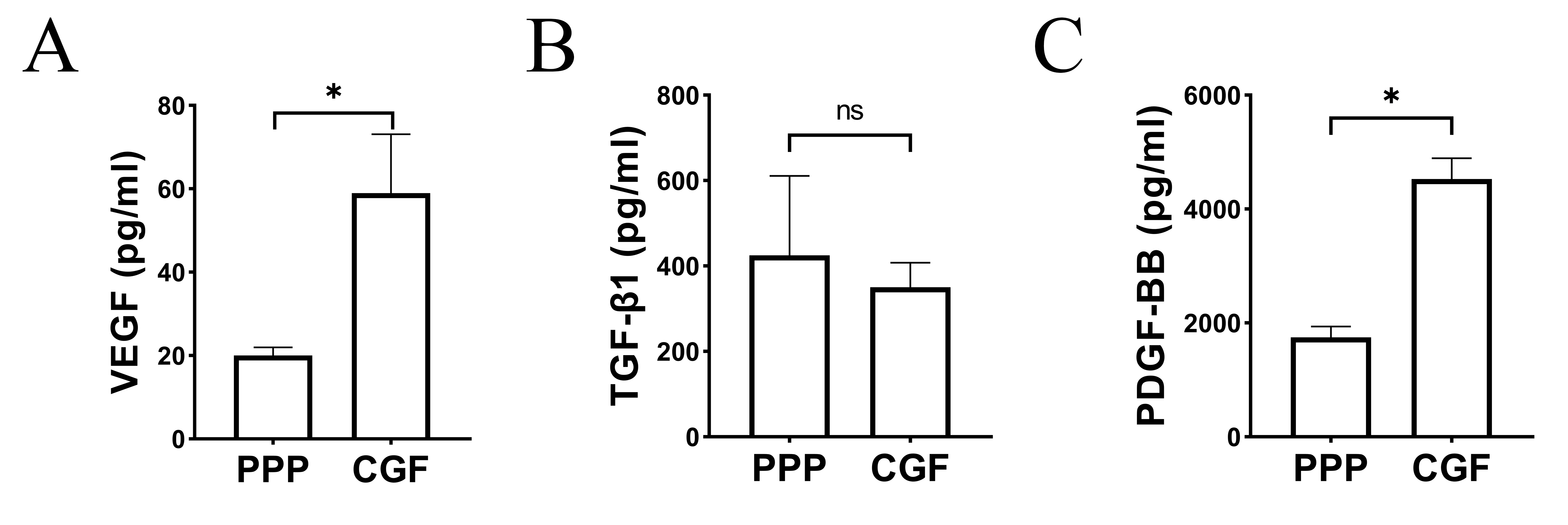

Supplement: Supplementary file 2 [file Image1.tif]
